# Deep Learning–Assisted Diagnosis of Cerebral Aneurysms Using the HeadXNet Model

**DOI:** 10.1001/jamanetworkopen.2019.5600

**Published:** 2019-06-07

**Authors:** Allison Park, Chris Chute, Pranav Rajpurkar, Joe Lou, Robyn L. Ball, Katie Shpanskaya, Rashad Jabarkheel, Lily H. Kim, Emily McKenna, Joe Tseng, Jason Ni, Fidaa Wishah, Fred Wittber, David S. Hong, Thomas J. Wilson, Safwan Halabi, Sanjay Basu, Bhavik N. Patel, Matthew P. Lungren, Andrew Y. Ng, Kristen W. Yeom

**Affiliations:** 1 Department of Computer Science, Stanford University, Stanford, California; 2 AIMI Center, Stanford University, Stanford, California; 3 Roam Analytics, San Mateo, California; 4 School of Medicine, Stanford University, Stanford, California; 5 School of Medicine, Department of Radiology, Stanford University, Stanford, California; 6 School of Medicine, Department of Neurosurgery, Stanford University, Stanford, California

## Abstract

**Importance:**

Deep learning has the potential to augment clinician performance in medical imaging interpretation and reduce time to diagnosis through automated segmentation. Few studies to date have explored this topic.

**Objective:**

To develop and apply a neural network segmentation model (the HeadXNet model) capable of generating precise voxel-by-voxel predictions of intracranial aneurysms on head computed tomographic angiography (CTA) imaging to augment clinicians’ intracranial aneurysm diagnostic performance.

**Design, Setting, and Participants:**

In this diagnostic study, a 3-dimensional convolutional neural network architecture was developed using a training set of 611 head CTA examinations to generate aneurysm segmentations. Segmentation outputs from this support model on a test set of 115 examinations were provided to clinicians. Between August 13, 2018, and October 4, 2018, 8 clinicians diagnosed the presence of aneurysm on the test set, both with and without model augmentation, in a crossover design using randomized order and a 14-day washout period. Head and neck examinations performed between January 3, 2003, and May 31, 2017, at a single academic medical center were used to train, validate, and test the model. Examinations positive for aneurysm had at least 1 clinically significant, nonruptured intracranial aneurysm. Examinations with hemorrhage, ruptured aneurysm, posttraumatic or infectious pseudoaneurysm, arteriovenous malformation, surgical clips, coils, catheters, or other surgical hardware were excluded. All other CTA examinations were considered controls.

**Main Outcomes and Measures:**

Sensitivity, specificity, accuracy, time, and interrater agreement were measured. Metrics for clinician performance with and without model augmentation were compared.

**Results:**

The data set contained 818 examinations from 662 unique patients with 328 CTA examinations (40.1%) containing at least 1 intracranial aneurysm and 490 examinations (59.9%) without intracranial aneurysms. The 8 clinicians reading the test set ranged in experience from 2 to 12 years. Augmenting clinicians with artificial intelligence–produced segmentation predictions resulted in clinicians achieving statistically significant improvements in sensitivity, accuracy, and interrater agreement when compared with no augmentation. The clinicians’ mean sensitivity increased by 0.059 (95% CI, 0.028-0.091; adjusted *P* = .01), mean accuracy increased by 0.038 (95% CI, 0.014-0.062; adjusted *P* = .02), and mean interrater agreement (Fleiss κ) increased by 0.060, from 0.799 to 0.859 (adjusted *P* = .05). There was no statistically significant change in mean specificity (0.016; 95% CI, −0.010 to 0.041; adjusted *P* = .16) and time to diagnosis (5.71 seconds; 95% CI, 7.22-18.63 seconds; adjusted *P* = .19).

**Conclusions and Relevance:**

The deep learning model developed successfully detected clinically significant intracranial aneurysms on CTA. This suggests that integration of an artificial intelligence–assisted diagnostic model may augment clinician performance with dependable and accurate predictions and thereby optimize patient care.

## Key Points

**Question:**

How does augmentation with a deep learning segmentation model influence the performance of clinicians in identifying intracranial aneurysms from computed tomographic angiography examinations?

**Findings:**

In this diagnostic study of intracranial aneurysms, a test set of 115 examinations was reviewed once with model augmentation and once without in a randomized order by 8 clinicians. The clinicians showed significant increases in sensitivity, accuracy, and interrater agreement when augmented with neural network model–generated segmentations.

**Meaning:**

This study suggests that the performance of clinicians in the detection of intracranial aneurysms can be improved by augmentation using deep learning segmentation models.

## Teaser

This diagnostic study investigates the use of a neural network segmentation model to assist clinicians in the detection of intracranial aneurisms.

## Introduction

Diagnosis of unruptured aneurysms is a critically important clinical task: intracranial aneurysms occur in 1% to 3% of the population and account for more than 80% of nontraumatic life-threatening subarachnoid hemorrhages.^1^ Computed tomographic angiography (CTA) is the primary, minimally invasive imaging modality currently used for diagnosis, surveillance, and presurgical planning of intracranial aneurysms,^2,3^ but interpretation is time consuming even for subspecialty-trained neuroradiologists. Low interrater agreement poses an additional challenge for reliable diagnosis.^4,5,6,7^

Deep learning has recently shown significant potential in accurately performing diagnostic tasks on medical imaging.^8^ Specifically, convolutional neural networks (CNNs) have demonstrated excellent performance on a range of visual tasks, including medical image analysis.^9^ Moreover, the ability of deep learning systems to augment clinician workflow remains relatively unexplored.^10^ The development of an accurate deep learning model to help clinicians reliably identify clinically significant aneurysms in CTA has the potential to provide radiologists, neurosurgeons, and other clinicians an easily accessible and immediately applicable diagnostic support tool.

In this study, a deep learning model to automatically detect intracranial aneurysms on CTA and produce segmentations specifying regions of interest was developed to assist clinicians in the interpretation of CTA examinations for the diagnosis of intracranial aneurysms. Sensitivity, specificity, accuracy, time to diagnosis, and interrater agreement for clinicians with and without model augmentation were compared.

## Methods

The Stanford University institutional review board approved this study. Owing to the retrospective nature of the study, patient consent or assent was waived. The Standards for Reporting of Diagnostic Accuracy (STARD) reporting guideline was used for the reporting of this study.

### Data

A total of 9455 consecutive CTA examination reports of the head or head and neck performed between January 3, 2003, and May 31, 2017, at Stanford University Medical Center were retrospectively reviewed. Examinations with parenchymal hemorrhage, subarachnoid hemorrhage, posttraumatic or infectious pseudoaneurysm, arteriovenous malformation, ischemic stroke, nonspecific or chronic vascular findings such as intracranial atherosclerosis or other vasculopathies, surgical clips, coils, catheters, or other surgical hardware were excluded. Examinations of injuries that resulted from trauma or contained images degraded by motion were also excluded on visual review by a board-certified neuroradiologist with 12 years of experience. Examinations with nonruptured clinically significant aneurysms (>3 mm) were included.^11^

### Radiologist Annotations

The reference standard for all examinations in the test set was determined by a board-certified neuroradiologist at a large academic practice with 12 years of experience who determined the presence of aneurysm by review of the original radiology report, double review of the CTA examination, and further confirmation of the aneurysm by diagnostic cerebral angiograms, if available. The neuroradiologist had access to all of the Digital Imaging and Communications in Medicine (DICOM) series, original reports, and clinical histories, as well as previous and follow-up examinations during interpretation to establish the best possible reference standard for the labels. For each of the aneurysm examinations, the radiologist also identified the location of each of the aneurysms. Using the open-source annotation software ITK-SNAP,^12^ the identified aneurysms were manually segmented on each slice.

### Model Development

In this study, we developed a 3-dimensional (3-D) CNN called HeadXNet for segmentation of intracranial aneurysms from CT scans. Neural networks are functions with parameters structured as a sequence of layers to learn different levels of abstraction. Convolutional neural networks are a type of neural network designed to process image data, and 3-D CNNs are particularly well suited to handle sequences of images, or volumes.

HeadXNet is a CNN with an encoder-decoder structure (eFigure 1 in the Supplement), where the encoder maps a volume to an abstract low-resolution encoding, and the decoder expands this encoding to a full-resolution segmentation volume. The segmentation volume is of the same size as the corresponding study and specifies the probability of aneurysm for each voxel, which is the atomic unit of a 3-D volume, analogous to a pixel in a 2-D image. The encoder is adapted from a 50-layer SE-ResNeXt network,^13,14,15^ and the decoder is a sequence of 3 × 3 transposed convolutions. Similar to UNet,^16^ skip connections are used in 3 layers of the encoder to transmit outputs directly to the decoder. The encoder was pretrained on the Kinetics-600 data set,^17^ a large collection of YouTube videos labeled with human actions; after pretraining the encoder, the final 3 convolutional blocks and the 600-way softmax output layer were removed. In their place, an atrous spatial pyramid pooling^18^ layer and the decoder were added.

### Training Procedure

Subvolumes of 16 slices were randomly sampled from volumes during training. The data set was preprocessed to find contours of the skull, and each volume was cropped around the skull in the axial plane before resizing each slice to 208 × 208 pixels. The slices were then cropped to 192 × 192 pixels (using random crops during training and centered crops during testing), resulting in a final input of size 16 × 192 × 192 per example; the same transformations were applied to the segmentation label. The segmentation output was trained to optimize a weighted combination of the voxelwise binary cross-entropy and Dice losses.^19^

Before reaching the model, inputs were clipped to [−300, 700] Hounsfield units, normalized to [−1, 1], and zero-centered. The model was trained on 3 Titan Xp graphical processing units (GPUs) (NVIDIA) using a minibatch of 2 examples per GPU. The parameters of the model were optimized using a stochastic gradient descent optimizer with momentum of 0.9 and a peak learning rate of 0.1 for randomly initialized weights and 0.01 for pretrained weights. The learning rate was scheduled with a linear warm-up from 0 to the peak learning rate for 10 000 iterations, followed by cosine annealing^20^ over 300 000 iterations. Additionally, the learning rate was fixed at 0 for the first 10 000 iterations for the pretrained encoder. For regularization, L2 weight decay of 0.001 was added to the loss for all trainable parameters and stochastic depth dropout^21^ was used in the encoder blocks. Standard dropout was not used.

To control for class imbalance, 3 methods were used. First, an auxiliary loss was added after the encoder and focal loss was used to encourage larger parameter updates on misclassified positive examples. Second, abnormal training examples were sampled more frequently than normal examples such that abnormal examples made up 30% of training iterations. Third, parameters of the decoder were not updated on training iterations where the segmentation label consisted of purely background (normal) voxels.

To produce a segmentation prediction for the entire volume, the segmentation outputs for sequential 16-slice subvolumes were simply concatenated. If the number of slices was not divisible by 16, the last input volume was padded with 0s and the corresponding output volume was truncated back to the original size.

### Study Design

**Figure 1.**
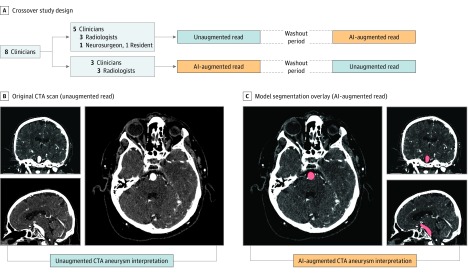
Study Design A, Crossover study design. Clinicians were divided into 2 groups to perform reads with and without model augmentation in random order, with a 2-week washout period between. B, Unaugmented read, with original CTA scan in axial, coronal, and sagittal view. C, Augmented read, with model segmentation overlay on CTA in axial, coronal, and sagittal view. Readers had the option to toggle overlays off and view the scan as shown in B. AI indicates artificial intelligence; CTA, computed tomographic angiography.

We performed a diagnostic accuracy study comparing performance metrics of clinicians with and without model augmentation. Each of the 8 clinicians participating in the study diagnosed a test set of 115 examinations, once with and once without assistance of the model. The clinicians were blinded to the original reports, clinical histories, and follow-up imaging examinations. Using a crossover design, the clinicians were randomly and equally divided into 2 groups. Within each group, examinations were sorted in a fixed random order for half of the group and sorted in reverse order for the other half. Group 1 first read the examinations without model augmentation, and group 2 first read the examinations with model augmentation. After a washout period of 14 days, the augmentation arrangement was reversed such that group 1 performed reads with model augmentation and group 2 read the examinations without model augmentation (Figure 1A).

Clinicians were instructed to assign a binary label for the presence or absence of at least 1 clinically significant aneurysm, defined as having a diameter greater than 3 mm. Clinicians read alone in a diagnostic reading room, all using the same high-definition monitor (3840 × 2160 pixels) displaying CTA examinations on a standard open-source DICOM viewer (Horos).^22^ Clinicians entered their labels into a data entry software application that automatically logged the time difference between labeling of the previous examination and the current examination.

When reading with model augmentation, clinicians were provided the model’s predictions in the form of region of interest (ROI) segmentations directly overlaid on top of CTA examinations. To ensure an image display interface that was familiar to all clinicians, the model’s predictions were presented as ROIs in a standard DICOM viewing software. At every voxel where the model predicted a probability greater than 0.5, readers saw a semiopaque red overlay on the axial, sagittal, and coronal series (Figure 1C). Readers had access to the ROIs immediately on loading the examinations, and the ROIs could be toggled off to reveal the unaltered CTA images (Figure 1B). The red overlays were the only indication that was given whether a particular CTA examination had been predicted by the model to contain an aneurysm. Given these model results, readers had the option to take it into consideration or disregard it based on clinical judgment. When readers performed diagnoses without augmentation, no ROIs were present on any of the examinations. Otherwise, the diagnostic tools were identical for augmented and nonaugmented reads.

### Statistical Analysis

On the binary task of determining whether an examination contained an aneurysm, sensitivity, specificity, and accuracy were used to assess the performance of clinicians with and without model augmentation. Sensitivity denotes the number of true-positive results over total aneurysm-positive cases, specificity denotes the number of true-negative results over total aneurysm-negative cases, and accuracy denotes the number of true-positive and true-negative results over all test cases. The microaverage of these statistics across all clinicians was also computed by measuring each statistic pertaining to the total number of true-positive, false-negative, and false-positive results. In addition, to convert the models’ segmentation output of the model into a binary prediction, a prediction was considered positive if the model predicted at least 1 voxel as belonging to an aneurysm and negative otherwise. The 95% Wilson score confidence intervals were used to assess the variability in the estimates for sensitivity, specificity, and accuracy.^23^

To assess whether the clinicians achieved significant increases in performance with model augmentation, a 1-tailed *t* test was performed on the differences in sensitivity, specificity, and accuracy across all 8 clinicians. To determine the robustness of the findings and whether results were due to inclusion of the resident radiologist and neurosurgeon, we performed a sensitivity analysis: we computed the *t* test on the differences in sensitivity, specificity, and accuracy across board-certified radiologists only.

The average time to diagnosis for the clinicians with and without augmentation was computed as the difference between the mean entry times into the spreadsheet of consecutive diagnoses; 95% *t* score confidence intervals were used to assess the variability in the estimates. To account for interruptions in the clinical read or time logging errors, the 5 longest and 5 shortest time to diagnosis for each clinician in each reading were excluded. To assess whether model augmentation significantly decreased the time to diagnosis, a 1-tailed *t* test was performed on the difference in average time with and without augmentation across all 8 clinicians.

The interrater agreement of clinicians and for the radiologist subset was computed using the exact Fleiss κ.^24^ To assess whether model augmentation increased interrater agreement, a 1-tailed permutation test was performed on the difference between the interrater agreement of clinicians on the test set with and without augmentation. The permutation procedure consisted of randomly swapping clinician annotations with and without augmentation so that a random subset of the test set that had previously been labeled as *read with augmentation* was now labeled as being *read without augmentation*, and vice versa; the exact Fleiss κ values (and the difference) were computed on the test set with permuted labels. This permutation procedure was repeated 10 000 times to generate the null distribution of the Fleiss κ difference (the interrater agreement of clinician annotations with augmentation is not higher than without augmentation) and the unadjusted *P *value calculated as the proportion of Fleiss κ differences that were higher than the observed Fleiss κ difference.

To control the familywise error rate, the Benjamini-Hochberg correction was applied to account for multiple hypothesis testing; a Benjamini-Hochberg–adjusted *P* ≤ .05 indicated statistical significance. All tests were 1-tailed.^25^

## Results

**Figure 2.**
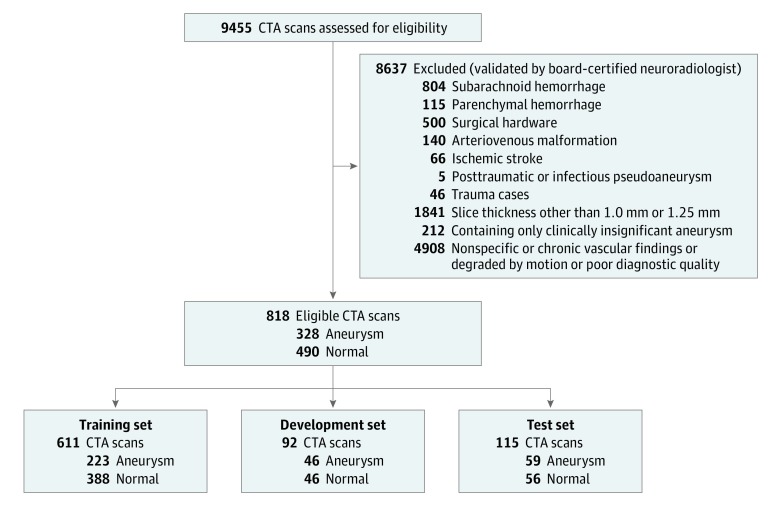
Data Set Selection Flow Diagram and Patient Demographics Of 9455 computed tomography angiogram (CTA) examinations performed between 2003 and 2017 at Stanford University Medical Center, 818 were selected according to an exclusion criteria validated by a board-certified neuroradiologist. These examinations were split into the training set, development set, and test set to be used for training models, selecting the best model, and assessing the selected model, respectively.

The data set contained 818 examinations from 662 unique patients with 328 CTA examinations (40.1%) containing at least 1 intracranial aneurysm and 490 examinations (59.9%) without intracranial aneurysms (Figure 2). Of the 328 aneurysm cases, 20 cases from 15 unique patients contained 2 or more aneurysms. One hundred forty-eight aneurysm cases contained aneurysms between 3 mm and 7 mm, 108 cases had aneurysms between 7 mm and 12 mm, 61 cases had aneurysms between 12 mm and 24 mm, and 11 cases had aneurysms 24 mm or greater. The location of the aneurysms varied according to the following distribution: 99 were located in the internal carotid artery, 78 were in the middle cerebral artery, 50 were cavernous internal carotid artery aneurysms, 44 were basilar tip aneurysms, 41 were in the anterior communicating artery, 18 were in the posterior communicating artery, 16 were in the vertebrobasilar system, and 12 were in the anterior cerebral artery. All examinations were performed either on a GE Discovery, GE LightSpeed, GE Revolution, Siemens Definition, Siemens Sensation, or a Siemens Force scanner, with slice thicknesses of 1.0 mm or 1.25 mm, using standard clinical protocols for head angiogram or head/neck angiogram. There was no difference between the protocols or slice thicknesses between the aneurysm and nonaneurysm examinations. For this study, axial series were extracted from each examination and a segmentation label was produced on every axial slice containing an aneurysm. The number of images per examination ranged from 113 to 802 (mean [SD], 373 [157]).

The examinations were split into a training set of 611 examinations (494 patients; mean [SD] age, 55.8 [18.1] years; 372 [60.9%] female) used to train the model, a development set of 92 examinations (86 patients; mean [SD] age, 61.6 [16.7] years; 59 [64.1%] female) used for model selection, and a test set of 115 examinations (82 patients; mean [SD] age, 57.8 [18.3] years; 74 [64.4%] female) to evaluate the performance of the clinicians when augmented with the model (Figure 2). Using stratified random sampling, the development and test sets were formed to include 50% aneurysm examinations and 50% normal examinations; the remaining examinations composed the training set, of which 36.5% were aneurysm examinations. Forty-three patients had multiple examinations in the data set due to examinations performed for follow-up of the aneurysm. To account for these repeat patients, examinations were split so that there was no patient overlap between the different sets. Figure 2 contains pathology and patient demographic characteristics for each set.

A total of 8 clinicians, including 6 board-certified practicing radiologists, 1 practicing neurosurgeon, and 1 radiology resident, participated as readers in the study. The radiologists’ years of experience ranged from 3 to 12 years, the neurosurgeon had 2 years of experience as attending, and the resident was in the second year of training at Stanford University Medical Center. Groups 1 and 2 consisted of 3 radiologists each; the resident and neurosurgeon were both in group 1. None of the clinicians were involved in establishing the reference standard for the examinations.

Without augmentation, clinicians achieved a microaveraged sensitivity of 0.831 (95% CI, 0.794-0.862), specificity of 0.960 (95% CI, 0.937-0.974), and an accuracy of 0.893 (95% CI, 0.872-0.912). With augmentation, the clinicians achieved a microaveraged sensitivity of 0.890 (95% CI, 0.858-0.915), specificity of 0.975 (95% CI, 0.957-0.986), and an accuracy of 0.932 (95% CI, 0.913-0.946). The underlying model had a sensitivity of 0.949 (95% CI, 0.861-0.983), specificity of 0.661 (95% CI, 0.530-0.771), and accuracy of 0.809 (95% CI, 0.727-0.870). The performances of the model, individual clinicians, and their microaverages are reported in eTable 1 in the Supplement.

**Figure 3.**
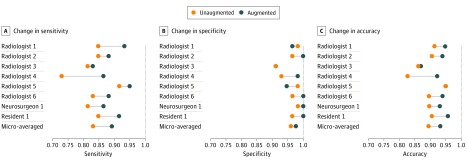
Change in Individual Clinicians' Performance Metric Horizontal lines depict the change in performance metric for each clinician with and without model augmentation. The orange dot represents performance without model, and the blue dot represents performance with model augmentation.

With augmentation, there was a statistically significant increase in the mean sensitivity (0.059; 95% CI, 0.028-0.091; adjusted *P* = .01) and mean accuracy (0.038; 95% CI, 0.014-0.062; adjusted *P* = .02) of the clinicians as a group. There was no statistically significant change in mean specificity (0.016; 95% CI, −0.010 to 0.041; adjusted *P* = .16). Performance improvements across clinicians are detailed in the Table, and individual clinician improvement in Figure 3. Individual performances with and without model augmentation are shown in eTable 1 in the Supplement. The sensitivity analysis confirmed that even among board-certified radiologists, there was a statistically significant increase in mean sensitivity (0.059; 95% CI, 0.013-0.105; adjusted *P* = .04) and accuracy (0.036; 95% CI, 0.001-0.072; adjusted *P* = .05). Performance improvements of board-certified radiologists as a group are shown in eTable 2 in the Supplement.

**Table.** Clinician Performance Metrics With and Without Augmentation

Metric	Microaverage (95% CI)		Mean Increase (95% CI)	*P* Value	
	Without Augmentation	With Augmentation		Unadjusted	Adjusted^a^
Sensitivity	0.831 (0.794 to 0.862)	0.890 (0.858 to 0.915)	0.059 (0.028 to 0.091)	.001	.01
Specificity	0.960 (0.937 to 0.974)	0.975 (0.957 to 0.986)	0.016 (−0.010 to 0.041)	.10	.16
Accuracy	0.893 (0.782 to 0.912)	0.932 (0.913 to 0.946)	0.038 (0.014 to 0.062)	.004	.02

^a^ *P* values were adjusted for multiple hypothesis testing using the Benjamini-Hochberg correction.

The mean diagnosis time per examination without augmentation microaveraged across clinicians was 57.04 seconds (95% CI, 54.58-59.50 seconds). The times for individual clinicians are detailed in eTable 3 in the Supplement, and individual time changes are shown in eFigure 2 in the Supplement. With augmentation, there was no statistically significant decrease in mean diagnosis time (5.71 seconds; 95% CI, −7.22 to 18.63 seconds; adjusted *P* = .19). The model took a mean of 7.58 seconds (95% CI, 6.92-8.25 seconds) to process an examination and output its segmentation map.

Confusion matrices, which are tables reporting true- and false-positive results and true- and false-negative results of each clinician with and without model augmentation, are shown in eTable 4 in the Supplement.

There was a statistically significant increase of 0.060 (adjusted *P* = .05) in the interrater agreement among the clinicians, with an exact Fleiss κ of 0.799 without augmentation and 0.859 with augmentation. For the board-certified radiologists, there was an increase of 0.063 in their interrater agreement, with an exact Fleiss κ of 0.783 without augmentation and 0.847 with augmentation.

## Discussion

In this study, the ability of a deep learning model to augment clinician performance in detecting cerebral aneurysms using CTA was investigated with a crossover study design. With model augmentation, clinicians’ sensitivity, accuracy, and interrater agreement significantly increased. There was no statistical change in specificity and time to diagnosis.

Given the potential catastrophic outcome of a missed aneurysm at risk of rupture, an automated detection tool that reliably detects and enhances clinicians’ performance is highly desirable. Aneurysm rupture is fatal in 40% of patients and leads to irreversible neurological disability in two-thirds of those who survive; therefore, an accurate and timely detection is of paramount importance. In addition to significantly improving accuracy across clinicians while interpreting CTA examinations, an automated aneurysm detection tool, such as the one presented in this study, could also be used to prioritize workflow so that those examinations more likely to be positive could receive timely expert review, potentially leading to a shorter time to treatment and more favorable outcomes.

The significant variability among clinicians in the diagnosis of aneurysms has been well documented and is typically attributed to lack of experience or subspecialty neuroradiology training, complex neurovascular anatomy, or the labor-intensive nature of identifying aneurysms. Studies have shown that interrater agreement of CTA-based aneurysm detection is highly variable, with interrater reliability metrics ranging from 0.37 to 0.85,^6,7,26,27,28^ and performance levels that vary depending on aneurysm size and individual radiologist experience.^4,6^ In addition to significantly increasing sensitivity and accuracy, augmenting clinicians with the model also significantly improved interrater reliability from 0.799 to 0.859. This implies that augmenting clinicians with varying levels of experience and specialties with models could lead to more accurate and more consistent radiological interpretations.

Currently, tools to improve clinician aneurysm detection on CTA include bone subtraction,^29^ as well as 3-D rendering of intracranial vasculature,^30,31,32^ which rely on application of contrast threshold settings to better delineate cerebral vasculature and create a 3-D–rendered reconstruction to assist aneurysm detection. However, using these tools is labor- and time-intensive for clinicians; in some institutions, this process is outsourced to a 3-D lab at additional costs. The tool developed in this study, integrated directly in a standard DICOM viewer, produces a segmentation map on a new examination in only a few seconds. If integrated into the standard workflow, this diagnostic tool could substantially decrease both cost and time to diagnosis, potentially leading to more efficient treatment and more favorable patient outcomes.

Deep learning has recently shown success in various clinical image-based recognition tasks. In particular, studies have shown strong performance of 2-D CNNs in detecting intracranial hemorrhage and other acute brain findings, such as mass effect or skull fractures, on CT head examinations.^33,34,35,36^ Recently, one study^10^ examined the potential role for deep learning in magnetic resonance angiogram–based detection of cerebral aneurysms, and another study^37^ showed that providing deep learning model predictions to clinicians when interpreting knee magnetic resonance studies increased specificity in detecting anterior cruciate ligament tears. To our knowledge, prior to this study, deep learning had not been applied to CTA, which is the first-line imaging modality for detecting cerebral aneurysms. Our results demonstrate that deep learning segmentation models may produce dependable and interpretable predictions that augment clinicians and improve their diagnostic performance. The model implemented and tested in this study significantly increased sensitivity, accuracy, and interrater reliability of clinicians with varied experience and specialties in detecting cerebral aneurysms using CTA.

### Limitations

This study has limitations. First, because the study focused only on nonruptured aneurysms, model performance on aneurysm detection after aneurysm rupture, lesion recurrence after coil or surgical clipping, or aneurysms associated with arteriovenous malformations has not been investigated. Second, since examinations containing surgical hardware or devices were excluded, model performance in their presence is unknown. In a clinical environment, CTA is typically used to evaluate for many types of vascular diseases, not just for aneurysm detection. Therefore, the high prevalence of aneurysm in the test set and the clinician’s binary task could have introduced bias in interpretation. Also, this study was performed on data from a single tertiary care academic institution and may not reflect performance when applied to data from other institutions with different scanners and imaging protocols, such as different slice thicknesses.

## Conclusions

A deep learning model was developed to automatically detect clinically significant intracranial aneurysms on CTA. We found that the augmentation significantly improved clinicians’ sensitivity, accuracy, and interrater reliability. Future work should investigate the performance of this model prospectively and in application of data from other institutions and hospitals.

## Notes

Supplement. **eFigure 1.** Diagram of Model Architecture **eFigure 2.** Individual Changes in Time Spent per Case **eTable 1.** Comparison of Individual Unaugmented and Augmented Clinicians in Aneurysm Detection on the Test Set **eTable 2.** Mean Increase in Board-Certified Radiologists’ Metrics as a Group **eTable 3.** Comparison of Individual Unaugmented and Augmented Clinicians in Time Spent on Aneurysm Detection on the Test Set **eTable 4.** Comparison of Confusion Matrices of Individual Unaugmented and Augmented Clinicians on Aneurysm Detection on the Test Set
