# Supplementary material for: Deep Learning–Assisted Diagnosis of Cerebral Aneurysms Using the HeadXNet Model
Source: JAMA Netw Open. 2019 Jun 7;2(6):e195600. doi: 10.1001/jamanetworkopen.2019.5600 (PMC6563570; doi:10.1001/jamanetworkopen.2019.5600)
Supplement: Supplement. — eFigure 1. Diagram of Model Architecture eFigure 2. Individual Changes in Time Spent per Case eTable 1. Comparison of Individual Unaugmented and Augmented Clinicians in Aneurysm Detection on the Test Set eTable 2. Mean Increase in Board-Certified Radiologists’ Metrics as a Group eTable 3. Comparison of Individual Unaugmented and Augmented Clinicians in Time Spent on Aneurysm Detection on the Test Set eTable 4. Comparison of Confusion Matrices of Individual Unaugmented and Augmented Clinicians on Aneurysm Detection on the Test Set [file jamanetwopen-2-e195600-s001.pdf]

Supplementary Online Content

Park A, Chute C, Rajpurkar P, et al. Deep learning–assisted diagnosis of cerebral aneurysms using the HeadXNet Model. *JAMA Netw Open*. 2019;2(6):e195600. doi:10.1001/jamanetworkopen.2019.5600

eFigure 1. Diagram of Model Architecture

eFigure 2. Individual Changes in Time Spent per Case

eTable 1. Comparison of Individual Unaugmented and Augmented Clinicians in Aneurysm Detection on the Test Set

eTable 2. Mean Increase in Board-Certified Radiologists' Metrics as a Group

eTable 3. Comparison of Individual Unaugmented and Augmented Clinicians in Time Spent on Aneurysm Detection on the Test Set

eTable 4. Comparison of Confusion Matrices of Individual Unaugmented and Augmented Clinicians on Aneurysm Detection on the Test Set

This supplementary material has been provided by the authors to give readers additional information about their work.

© 2019 Park A et al. *JAMA Network Open.*

eFigure 1. Diagram of Model Architecture.

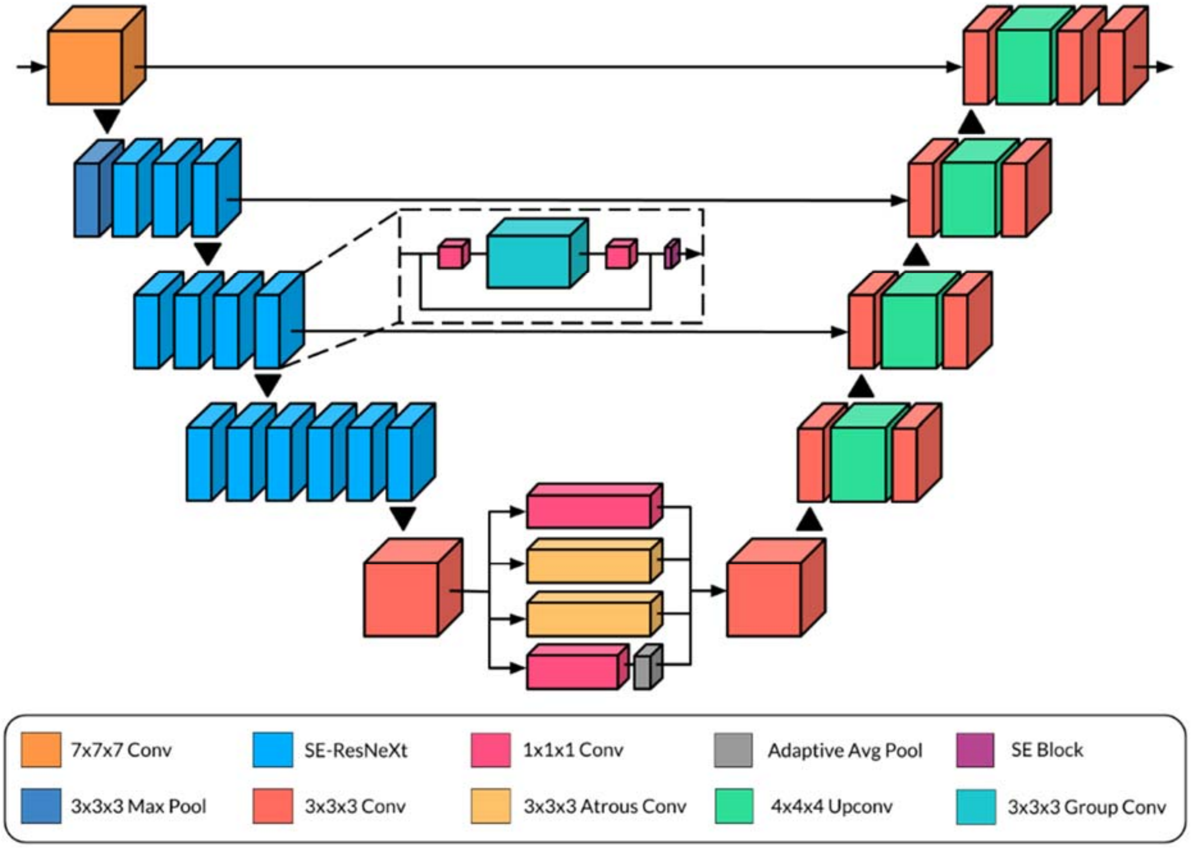

HeadXNet is a convolutional neural network (CNN) that takes in an input of a series of CTA images and outputs segmentation that consists of predicted probability of diagnosis for each voxel.

© 2019 Park A et al. *JAMA Network Open.*

eFigure 2. Individual Changes in Time Spent per Case.

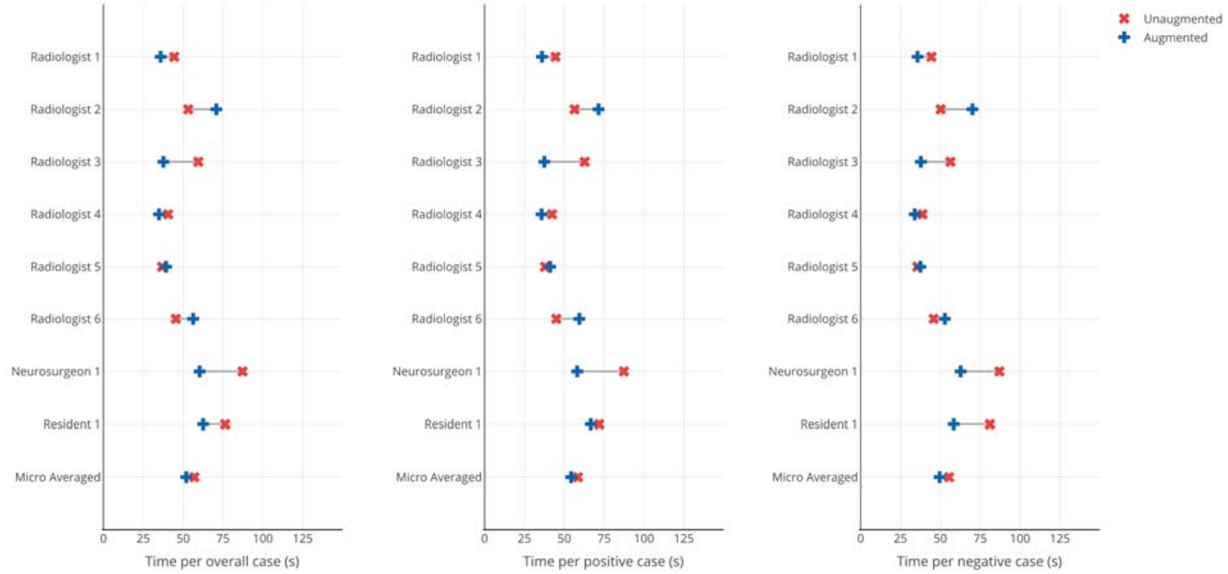

Bold grey lines depict the change in average time to diagnosis in seconds per case for each clinician with and without model augmentation. The red cross represents performance without model augmentation, and the blue plus represents performance with model augmentation.

© 2019 Park A et al. *JAMA Network Open.*

eTable 1. Comparison of Individual Unaugmented and Augmented Clinicians in Aneurysm Detection on the Test Set.

**Clinician**		**Augmentation**	**Sensitivity (95% CI)**	**Specificity (95% CI)**	**Accuracy (95% CI)**
**Radiologist 1**		Unaugmented	0.847 (0.735, 0.918)	0.982 (0.906, 0.997)	0.913 (0.847, 0.952)
		Augmented	0.932 (0.838, 0.973)	0.964 (0.879, 0.990)	0.948 (0.891, 0.976)
**Radiologist 2**		Unaugmented	0.847 (0.735, 0.918)	0.964 (0.879, 0.990)	0.904 (0.837, 0.946)
		Augmented	0.881 (0.775, 0.941)	1.000 (0.936, 1.000)	0.939 (0.880, 0.970)
**Radiologist 3**		Unaugmented	0.814 (0.696, 0.893)	0.911 (0.807, 0.961)	0.861 (0.786, 0.913)
		Augmented	0.831 (0.715, 0.905)	0.911 (0.807, 0.961)	0.870 (0.796, 0.919)
**Radiologist 4**		Unaugmented	0.729 (0.604, 0.826)	0.929 (0.830, 0.972)	0.826 (0.747, 0.885)
		Augmented	0.864 (0.755, 0.930)	0.982 (0.906, 0.997)	0.922 (0.858, 0.958)
**Radiologist 5**		Unaugmented	0.915 (0.816, 0.963)	0.982 (0.906, 0.997)	0.948 (0.891, 0.976)
		Augmented	0.949 (0.861, 0.983)	0.946 (0.854, 0.982)	0.948 (0.891, 0.976)
**Radiologist 6**		Unaugmented	0.831 (0.715, 0.905)	0.964 (0.879, 0.990)	0.896 (0.826, 0.939)
		Augmented	0.881 (0.775, 0.941)	1.000 (0.936, 1.000)	0.939 (0.880, 0.970)
**Neurosurgeon 1**		Unaugmented	0.814 (0.696, 0.893)	0.982 (0.906, 0.997)	0.896 (0.826, 0.939)
		Augmented	0.864 (0.755, 0.930)	1.000 (0.936, 1.000)	0.930 (0.869, 0.964)
**Resident 1**		Unaugmented	0.847 (0.735, 0.918)	0.964 (0.879, 0.990)	0.904 (0.837, 0.946)
		Augmented	0.915 (0.816, 0.963)	1.000 (0.936, 1.000)	0.957 (0.902, 0.981)
**Micro-average**		Unaugmented	0.831 (0.794, 0.862)	0.960 (0.937, 0.974)	0.893 (0.782, 0.912)
		Augmented	0.890 (0.858, 0.915)	0.975 (0.957, 0.986)	0.932 (0.913, 0.946)
	Model		0.949 (0.860, 0.963)	0.661 (0.530, 0.771)	0.809 (0.727, 0.870)

Sensitivity, specificity, and accuracy for each clinician, their micro-average, and the support model is shown. Confidence intervals were computed using 95% Wilson score confidence intervals to estimate the variability in each metric and are shown in parentheses.

© 2019 Park A et al. *JAMA Network Open.*

eTable 2. Mean Increase in Board-certified Radiologists' Metrics as a Group.

**Metric**	**Mean Increase (95% CI)**	**P-value (unadjusted)**	**P-value (adjusted)**
**Sensitivity**	0.059 (0.013, 0.105)	0.011	0.035
**Specificity**	0.012 (-0.025, 0.049)	0.222	0.222
**Accuracy**	0.036 (0.001, 0.072)	0.023	0.046

Controlling for multiple testing, adjusted p-values were computed using Benjamini-Hochberg correction. At the significance level of 0.05, the adjusted p-values show that the improvement in sensitivity and accuracy after model augmentation were statistically significant.

© 2019 Park A et al. *JAMA Network Open.*

eTable 3. Comparison of Individual Unaugmented and Augmented Clinicians in Time Spent on Aneurysm Detection on the Test Set.

Clinician	Augmentation	Overall cases (s) (95% CI)	Positive cases (s) (95% CI)	Negative cases (s) (95% CI)
**Radiologist 1**	Unaugmented	44.438 (40.727, 48.149)	44.472 (39.489, 49.455)	44.404 (38.722, 50.086)
	Augmented	35.857 (33.327, 38.387)	35.981 (32.336, 39.626)	35.736 (32.102, 39.369)
**Radiologist 2**	Unaugmented	53.143 (46.443, 59.843)	56.400 (46.410, 66.390)	50.182 (40.941, 59.423)
	Augmented	70.829 (64.043, 77.615)	71.491 (62.184, 80.797)	70.154 (59.947, 80.360)
**Radiologist 3**	Unaugmented	59.457 (53.669, 65.245)	62.765 (54.110, 71.419)	56.333 (48.416, 64.250)
	Augmented	37.629 (34.596, 40.662)	37.491 (33.173, 41.809)	37.769 (33.362, 42.177)
**Radiologist 4**	Unaugmented	40.524 (37.246, 43.801)	42.333 (37.682, 46.985)	38.815 (34.098, 43.532)
	Augmented	34.886 (32.231, 37.541)	35.745 (32.640, 38.851)	33.940 (29.426, 38.454)
**Radiologist 5**	Unaugmented	36.752 (34.480, 39.025)	37.855 (34.550, 41.159)	35.540 (32.360, 38.720)
	Augmented	39.257 (35.896, 42.618)	41.115 (36.490, 45.740)	37.434 (32.460, 42.408)
**Radiologist 6**	Unaugmented	45.390 (42.405, 48.376)	44.981 (40.723, 49.240)	45.808 (41.481, 50.134)
	Augmented	56.295 (51.457, 61.133)	59.436 (52.791, 66.081)	52.840 (45.665, 60.015)
**Neurosurgeon 1**	Unaugmented	87.219 (80.023, 94.415)	87.370 (77.661, 97.079)	87.059 (76.040, 98.077)
	Augmented	60.314 (54.901, 65.728)	58.091 (50.977, 65.204)	62.760 (54.300, 71.220)
**Resident 1**	Unaugmented	76.371 (69.131, 83.612)	71.889 (63.328, 80.449)	81.118 (69.105, 93.130)
	Augmented	62.571 (57.068, 68.074)	66.660 (58.406, 74.915)	58.404 (51.041, 65.766)
**Micro-average**	Unaugmented	57.040 (54.575, 59.504)	58.535 (55.094, 61.975)	55.498 (51.958, 59.037)
	Augmented	51.973 (49.725, 54.220)	54.258 (50.929, 57.586)	49.574 (46.575, 52.574)
**Model**		7.581 (6.916, 8.246)	7.642 (6.646, 8.637)	7.519 (6.606, 8.433)

Average time to diagnosis in seconds per case for overall cases, positive cases, and negative cases is shown for each clinician. Confidence intervals were computed using *t*-score confidence intervals to estimate the variability in each metric and are shown in brackets.

© 2019 Park A et al. *JAMA Network Open.*

eTable 4. Comparison of Confusion Matrices of Individual Unaugmented and Augmented Clinicians on Aneurysm Detection on the Test Set.

Clinician	Augmentation	True Positives	False Positives	True Negatives	False Negatives
**Radiologist 1**	Unaugmented	55	1	9	50
	Augmented	54	2	4	55
**Radiologist 2**	Unaugmented	54	2	9	50
	Augmented	56	0	7	52
**Radiologist 3**	Unaugmented	51	5	11	48
	Augmented	51	5	10	49
**Radiologist 4**	Unaugmented	52	4	16	43
	Augmented	55	1	8	51
**Radiologist 5**	Unaugmented	55	1	5	54
	Augmented	53	3	3	56
**Radiologist 6**	Unaugmented	54	2	10	49
	Augmented	56	0	7	52
**Neurosurgeon 1**	Unaugmented	55	1	11	48
	Augmented	56	0	8	51
**Resident 1**	Unaugmented	54	2	9	50
	Augmented	56	0	5	54
**Micro-average**	Unaugmented	430	18	80	392
	Augmented	437	11	52	420
**Model**		37	19	3	56

Number of true positive, false positive, true negative, and false negative diagnoses out of the 115 test cases for each clinician and the model is shown.
